# FBX aqueous chemical dosimeter for measurement of virtual wedge profiles

**DOI:** 10.1120/jacmp.v9i4.2809

**Published:** 2008-10-24

**Authors:** Manoj K. Semwal, Anil K. Bansal, Pradeep K. Thakur, Pandit B. Vidyasagar

**Affiliations:** ^1^ Department of Radiotherapy All India Institute of Medical Sciences New Delhi; ^2^ Army Hospital Research and Referral Department of Radiotherapy, All India Institute of Medical Sciences New Delhi; ^3^ Department of Radiotherapy University of Pune Pune India; ^4^ Command Hospital (Southern Command), and Department of Physics University of Pune Pune India

**Keywords:** FBX dosimeter, virtual wedge, profile measurement

## Abstract

We investigated the ferrous sulfate–benzoic acid–xylenol orange (FBX) aqueous chemical dosimeter for measurement of virtual (dynamic) wedge profiles on a linear accelerator. The layout for irradiation of the FBX‐filled tubes mimicked a conventional linear detector array geometry. A comparison of the resulting measurements with film‐measured profiles showed that, in the main beam region, the difference between the FBX system and the film system was within ±2% and that, in the penumbra region, the difference varied from ±1mm to ±2.5mm in terms of positional equivalence, depending on the size of the dosimeter tubes. We thus believe that the energy‐independent FBX dosimetry system can measure virtual wedge profiles with reasonable accuracy at reasonable cost. However, efficiency improvement is required before this dosimetry system can be accepted into routine practice.

PACS numbers: 87.53.‐j, 87.53.Dq, 87.53.Mr, 87.53.Xd, 87.66.Ff

## I. INTRODUCTION

The ferrous sulfate–benzoic acid–xylenol orange (FBX) aqueous chemical dosimetry system was developed as a modification of the Fricke system by Gupta and co‐workers in the 1970s for low‐level measurements.^(1)^ ^,^ ^(2)^ In the Fricke system, the absorbed dose is measured in terms of oxidation yield of ferric ions produced by irradiation of the FBX solution. The ferric ions form a color complex with xylenol orange, resulting in a color change of the solution. This change in color is measured spectrophotometrically at about 540 nm as an estimate of the ferric ion yield. This system has been studied for various applications in radiotherapy such as percentage depth dose measurements, machine output calibrations,^(^ ^3^ ^–^ ^5^ ^)^ brachytherapy source calibration, and in vivo dosimetry.^(6)^ ^,^ ^(7)^ One of the important properties of this dosimeter is its energy‐independent response for X‐rays and gamma rays from 33 keV to 1250 keV.^(1)^

In the study presented here, we used the FBX system to measure virtual wedge (VW) profiles by making arrays of FBX‐filled tubes. We then compared the FBX measurements with film measurements. Our goal in this investigation was to determine whether the energy‐independent and cost‐effective FBX system could be used for VW dosimetry as an alternative to commonly used systems such as films or linear detector arrays (LDAs) of diodes or ion chambers.

## II. MATERIALS AND METHODS

The FBX dosimeter solution was prepared following the method described by Gupta et al.^(3)^ to achieve the following composition: $0.2\;\text{mol}m^{-3}$ ferrous ammonium sulfate, $5.0\;\text{mol}m^{-3}$ benzoic acid, and $0.20\;\text{mol}m^{-3}$ xylenol orange in $40.0\;\text{mol}m^{-3}$ sulfuric acid. All chemicals came from E. Merck (Darmstadt, Germany), except for the xylenol orange, which came from Loba Chemie (Vienna, Austria).

For irradiation purpose, tubes of three different sizes were used to prepare FBX dosimeter arrays:

- Stoppered polypropylene tubes (PTs) of approximately 1.4 cm outer diameter, 5.5 cm length, and 0.1 cm wall thickness available in the market
- Tubes made from the plastic needle covers of 5 mL disposable syringes with modified needle bases as stoppers [the outer diameter of these cylindrical needle cover tubes (NCTs) was approximately 0.6 cm, and the length, 5 cm]
- Tubes made from intravenous‐drip catheters (IVTs) by heat‐sealing at both ends (the outer diameter of the IVTs was approximately 0.4 cm, and the length, approximately 5.0 cm)

**Figure 1:**
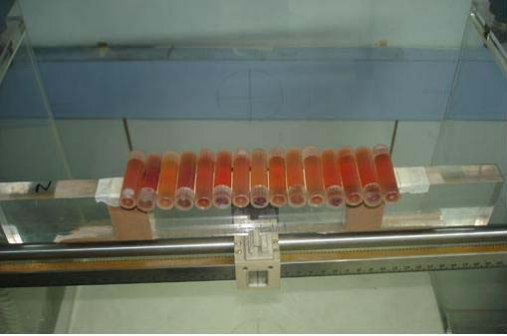
Dosimeter tube array setup. Polypropylene tubes filled with FBX (ferrous sulfate–benzoic acid–xylenol orange) solution arranged as an array on a polymethyl methacrylate slab placed in a water phantom.

The FBX‐filled tubes were arranged in the form of an array, imitating an LDA setup. The tubes were placed horizontally (15 tubes in the case of PTs, 21 tubes in the case of NCTs, and 25 tubes in the case of IVTs) on a polymethyl methacrylate (PMMA) slab, which in turn was placed in a water phantom as shown in Fig. 1. The long axes of the tubes were placed perpendicular to the long axis of the PMMA slab.

The IVTs were irradiated in a Virtual Water solid phantom with 0.5 cm jelly bolus (both from Med‐Tec, Orange City, IA) immediately above and below. Bolus was used to minimize the air gaps between adjacent tubes. In addition, wet cotton was used to fill any remaining gap between the tubes. The PTs, being larger in size, were placed without spacing between them. The NCTs and the IVTs were placed 1 cm apart over the central 6 cm of the field and adjacent beyond this region to a distance of about 4 cm on each side, so as to cover the steep dose gradient region. The final position of each tube with respect to the central tube was noted after tube placement was complete.

A Siemens Primus linear accelerator (Siemens Medical Systems, Concord, CA) was used for irradiation. The non–multileaf collimator upper jaws (Y jaws) of the secondary collimator in this accelerator can be used for delivery of VW treatment. We chose a 6 MV photon beam and a 45‐degree VW with a field size of 10×10 cm at a source‐to‐surface distance of 100 cm for this study as a typical case. The tube arrays were irradiated at 1.5 cm, 10 cm, and 20 cm depths with a dose of 200 cGy delivered to the central axis at each depth. The collimator rotation was such that the moving jaw during the operation of the VW was along the length of the PMMA slab.

The optical density (OD) measurements for the PTs and the NCTs were carried out in a digital 5‐filter colorimeter (Aimil, New Delhi, India) at 540 nm. Because the solution quantity available for the IVTs (about 200 μL) was insufficient for colorimetric measurement, a Hitachi U 3300 spectrophotometer Hitachi, Tokyo, Japan) having a 50 μL cuvette was used for OD measurements at 548 nm.

To compare the performance of the FBX system with film measurements, Kodak X‐Omat V radiotherapy verification ready‐pack films (Eastman Kodak Company, Rochester, NY) were also used for profile measurements in the solid phantom with similar irradiation geometry. The dose delivered in this case was 100 cGy. A standard procedure as mentioned by Elder et al.^(8)^ for film irradiation, processing, and calibration was followed. The films were read on a WP102 densitometer (Wellhofer Dosimetrie, Nuremburg, Germany) with 2 mm spatial resolution using WP700 application software. The film profiles were, in turn, validated with ion‐chamber (IC15: Wellhofer Dosimetrie) measurements for open fields at 1.5 cm $\left(d_{\max}\right)$ and 10.0 cm depths in the water phantom. Our results show that the absolute difference in the percent dose (dose relative to $D_{\max}$) is within ±2% at both depths for the film and for the ion chamber. This finding agrees with the results published by Liu et al.^(9)^

## III. RESULTS AND DISCUSSION

**Figure 2:**
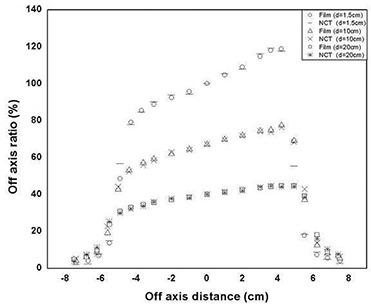
Virtual wedge profiles measured with film and with an FBX (ferrous sulfate–benzoic acid–xylenol orange) dosimeter array. The off‐axis ratio values were measured with needle‐cover tubes (NCTs) and film in a 6 MV photon beam. The depths for measurement were 1.5 cm, 10.0 cm, and 20.0 cm at a source‐to‐surface distance of 100 cm for a field size of 10×10 cm.

Fig. 2 shows the off‐axis ratio profiles measured with the NCTs and the film for a 45‐degree VW at 1.5 cm, 10 cm, and 20 cm depths with normalization at the central axis for the $d_{\max}$ position. The off‐axis ratio values for film were estimated for the corresponding FBX tube positions with respect to the central axis. The results show that, in the main beam region, the film and the FBX‐measured off‐axis ratio values agree closely regardless of tube size, but toward the beam edges, the difference increases with the size of the tubes in the FBX array. In the main beam region, the differences in absolute off‐axis ratio values for the PT array and the film were within ±1%, and in the penumbral region, the average difference in terms of positional error was ±2mm, with a maximum value of 2.5 mm. In the case of the NCT array, the corresponding differences with the film measurements were within ±1.5% and ±1.5mm; for the IVT array, they were within ±1.9% and ±1.0mm.

The good agreement between the FBX and film measurements in the main beam region regardless of tube size could be explained by the fact that, in this region, the dose gradient is gradual and almost linear. Hence, the size of the tubes is not important. In the penumbra region where the dose gradient is steep, the increased difference between the film and FBX measurements is related mainly to tube size, the larger tubes having lower spatial resolution. Further, the random errors associated with the measurements were greatest with the smallest tube size (±1.7% for the IVTs) and least with the largest tube size (±0.7% for PTs). That finding could be attributed to the larger errors introduced during the handling of considerably smaller quantities of FBX solution (about 200 μL) in the case of IVTs as compared with about 1.5 mL in the case of PTs. We believe that, with the present setup, about 0.5 mL is the optimum quantity of FBX solution for good results and that a further decrease in tube size to improve spatial resolution could be offset by the increase in overall uncertainty of the measurement.

## IV. CONCLUSIONS

The results indicate that the FBX dosimeter, with a colorimeter for OD measurements, can measure VW profiles with the reasonable accuracy needed for routine quality assurance purposes. However, using the current technique, it takes approximately 5.5 hours to measure profiles at three depths for one field size, as compared with the 2 hours needed for film measurement. Hence, an improvement in efficiency is required before the FBX system can be used in a clinical setting. We believe that there is room for improvement in the efficiency of our FBX dosimetry procedure, given that we used crude in‐house accessories that were basically meant to validate the system. The availability of tubes with easier and quicker solution filling and extraction mechanisms, and some technique improvement in the preparation of the arrays will reduce the time considerably. The cost of setting up a FBX dosimetry facility is approximately US$450, comprising mainly chemicals and glassware (US$300) and the colorimeter (US$150). Once purchased, the chemicals can last for several years depending on use, and the cost of the chemicals and consumables required for preparing the 500 mL FBX solution needed for three profile measurements is estimated at about US$1. The other attractive features of the FBX system, as mentioned earlier, are its water‐equivalent composition and its energy‐independent response for X rays and gamma rays (unlike the response of films and ion chambers).
